# Death of Sir Spencer Wells

**Published:** 1897-02-06

**Authors:** 


### Death of Sir Spencer Wells.

We regret to have to record the death of Sir Spencer Wells, which took place at Antibes last Sunday. Sir Spencer Wells was a man of many parts and with wide interests, but he will always be known and remembered for his work in rehabilitating and bringing into popular and professional recognition the operation of ovariotomy. Sir Spencer Wells was born at St. Albans on February 3rd, 1818, and he became a member of the Royal College of Surgeons in 1841.

Entering the Navy as an assistant-surgeon, he spent six years at Malta; and then, in 1853, commenced practice in London, becoming attached to the Samaritan Hospital, which at that time was but a dispensary, the following year. In 1854 he was sent out by the Secretary for War as Chief Surgeon at Smyrna and Rankioi, and on the termination of the war he returned to London.

Before going to Smyrna in 1854 he had assisted Mr. Baker Brown in an ovariotomy at the Middlesex Hospital, which, however, had ended fatally; but while in the East he was much struck by the impunity with which in many cases injuries involving the peritoneum were recovered from, and he was led to think again of the possibility of obtaining a greater success in the operation of ovariotomy than had hitherto attended it. On his return to London he took up his work again at the Samaritan, and soon began to do ovariotomy, with at first only a moderate success. During the session of 1859-60, however, he was able to report a series of one hundred cases to the Royal Medical and Chirurgical Society. The success which he had obtained was great, and from that time the position of the operation was secured, and the name of Spencer Wells was firmly written on the roll of English worthies.

Sir Spencer Wells was elected on the council of the College of Surgeons in 1871, and became president in 1882. The baronetcy was conferred upon him in 1883, and he has been the recipient of many honours from abroad. For many years he held the office of Surgeon to the Queen's Household.

In his earlier years he was for some time editor of the *Medical Times and Gazette*. He had latterly taken great interest in sanitary matters, and had busied himself much with advocating the cremation of the dead.

Sir Spencer Wells leaves a son, Mr. Arthur Spencer Wells, who was born in 1866, and to whom the baronetcy descends, and two daughters.

